# Quantifying the Contribution of Entire Free-Living Nematode Communities to Carbon Mineralization under Contrasting C and N Availability

**DOI:** 10.1371/journal.pone.0136244

**Published:** 2015-09-22

**Authors:** Mesfin Tsegaye Gebremikael, Hanne Steel, Wim Bert, Peter Maenhout, Steven Sleutel, Stefaan De Neve

**Affiliations:** 1 Department of Soil Management, University of Ghent, Ghent, Belgium; 2 Department of Biology, Nematology Research Unit, University of Ghent, Ghent, Belgium; Chinese Academy of Sciences, CHINA

## Abstract

To understand the roles of nematodes in organic matter (OM) decomposition, experimental setups should include the entire nematode community, the native soil microflora, and their food sources. Yet, published studies are often based on either simplified experimental setups, using only a few selected species of nematode and their respective prey, despite the multitude of species present in natural soil, or on indirect estimation of the mineralization process using O_2_ consumption and the fresh weight of nematodes. We set up a six-month incubation experiment to quantify the contribution of the entire free living nematode community to carbon (C) mineralization under realistic conditions. The following treatments were compared with and without grass-clover amendment: defaunated soil reinoculated with the entire free living nematode communities (+Nem) and defaunated soil that was not reinoculated (-Nem). We also included untreated fresh soil as a control (CTR). Nematode abundances and diversity in +Nem was comparable to the CTR showing the success of the reinoculation. No significant differences in C mineralization were found between +Nem and -Nem treatments of the amended and unamended samples at the end of incubation. Other related parameters such as microbial biomass C and enzymatic activities did not show significant differences between +Nem and -Nem treatments in both amended and unamended samples. These findings show that the collective contribution of the entire nematode community to C mineralization is small. Previous reports in literature based on simplified experimental setups and indirect estimations are contrasting with the findings of the current study and further investigations are needed to elucidate the extent and the mechanisms of nematode involvement in C mineralization.

## Introduction

Organic matter (OM) decomposition processes and the three primary energy pathways that exist in the soil food web, i.e., the bacterial, fungal and root channels are regulated by free-living nematodes [1–3]. For example, the fastest decomposition pathway in which bacteria are the primary decomposers, OM decomposition is affected by bacterivorous and omnivorous nematodes as they feed on and disperse these microbes [4–6]. Plant-root feeding and fungi feeding nematodes regulate the root and fungal energy channels, respectively. As they pierce the cell wall of plants or hyphae, part of the cell contents is transferred to and increases the size of the labile organic matter pool [2] which is a rate limiting factor for C and N mineralization [7, 8]. Two main mechanisms have been reported how nematodes are involved in C and N mineralization and nutrient flow in the soil food web: 1) C:N ratio differences between nematodes and their prey, particularly bacteria [6, 9, 10] and 2) high respiration efficiencies, wherein most of the assimilated C is respired as CO_2_ [6, 11–13].

Respiration efficiencies, defined as the amount of C respired per unit of C assimilated, have been consistently reported to be high (60–85%) for several nematode species in various feeding groups [11], indicating that it may be the main mechanism how nematodes are involved in energy and nutrient flows in soils. Despite such high respiration efficiency, previous studies reported that the contribution of nematodes to the total soil heterotrophic respiration is low. For instance, [14] estimated that nematodes contribute 0.8% and [15] 2% of the total heterotrophic soil respiration in a Japanese coniferous and US deciduous forest, respectively. These respiration values are not often determined by measuring CO_2_ experimentally, but calculated using the relation between the estimated weight of nematode population, their oxygen consumption and estimated values of the respiration quotient (RQ) [16]. Due to these methodological limitations, the reported low contribution of nematodes to the total heterotrophic soil respiration may thus be questioned.

In contrast to the above estimates, studies which directly measured the relative CO_2_ respired by nematodes in soil microcosms reported significantly higher contributions of nematodes to C mineralization. For instance, [17] found that the presence of a bacterivorous nematode increased cumulative CO_2_-C mineralized by 50% and 27% over the presence of bacteria only in glucose amended and unamended treatments, respectively. While such kinds of experiments based on a single species of nematodes and bacteria are useful in investigating specific mechanisms in detail, the findings cannot be considered as the effect of the entire nematode community. Such findings obviously are not representative of field conditions where many species of nematodes interact amongst each other and with a multitude species of microbes. Moreover, the contribution of individual species to nutrient cycling may vary considerably as each nematode taxon exhibits a wide range of metabolic activity, and their energetics may depend on the food availability (C and N) and the nematode growth stage [18].

Thus, to obtain reliable estimates, the contribution of the entire nematode community to C mineralization needs to be determined in an experimental setup that allow variations in C and N availability and all interactions between different species of nematodes and native microbes. However, to the best of our knowledge, there are no data on the collective contribution of total nematode populations to C mineralization based on measured differences in CO_2_-C production between treatments with and without nematodes. To do so, we set up an incubation experiment and measured CO_2_ evolution in defaunated soil cores with or without nematodes for six months in both unamended and grass-clover amended soils. The main objective of this experiment was to quantify the contribution of the entire free-living soil nematode community to C mineralization from indigenous soil organic matter and grass-clover amendment under a relatively realistic experimental setup. We hypothesized that the presence of the entire free-living nematode community would collectively enhance C mineralization through direct release of CO_2_ and indirectly through increased microbial turnover in comparison to their absence in both amended and unamended soils.

## Materials and Methods

### 2.1 Sample collection and preparation

Composite soil samples were collected from the 0-15cm layer of an organically managed agricultural experimental field in Merelbeke, Belgium, sown with a grass-clover mix. The field is owned by the Flemish Institute for Agricultural and Fisheries Research (ILVO) and a permission to collect the sample was granted by ILVO. Several augerings were collected in a zigzag pattern throughout the field after making plant free patches by gently cutting the grass-clover at the base of the plants. At the time of sampling the soil was characterized by a bulk density of 1.49 Mg m^-3^, 1.23% organic C, 0.10% total N, and 22.3 mg mineral N kg^-1^ dry soil. A bulk sample of fresh grass-clover was also collected from the same field, chopped into small pieces and used as organic amendment in the experiment.

The composite sample was gently sieved through a 5 mm mesh in order to homogenize it and to remove stones and visible soil animals such as earthworms. Part of the composite soil sample (about 16 kg fresh weight) was kept separately for nematode extraction. Part of the composite soil was transferred into four large sized PVC columns (30 x 10.8 cm) each containing about 3 kg fresh soil. An appropriate amount of chopped fresh grass-clover was weighed and mixed into two of the PVC columns that were randomly assigned to amended treatments at a rate of 4.96 tons DM ha^-1^(the rate determined based on the grass-clover yield of the field and N recommendation for the grass production). The remaining two PVC columns that did not receive grass-clover were used for unamended treatments. The soil samples in each of the four PVC columns were packed at a bulk density of 1.4 Mg m^-3^.

**Fig 1:**
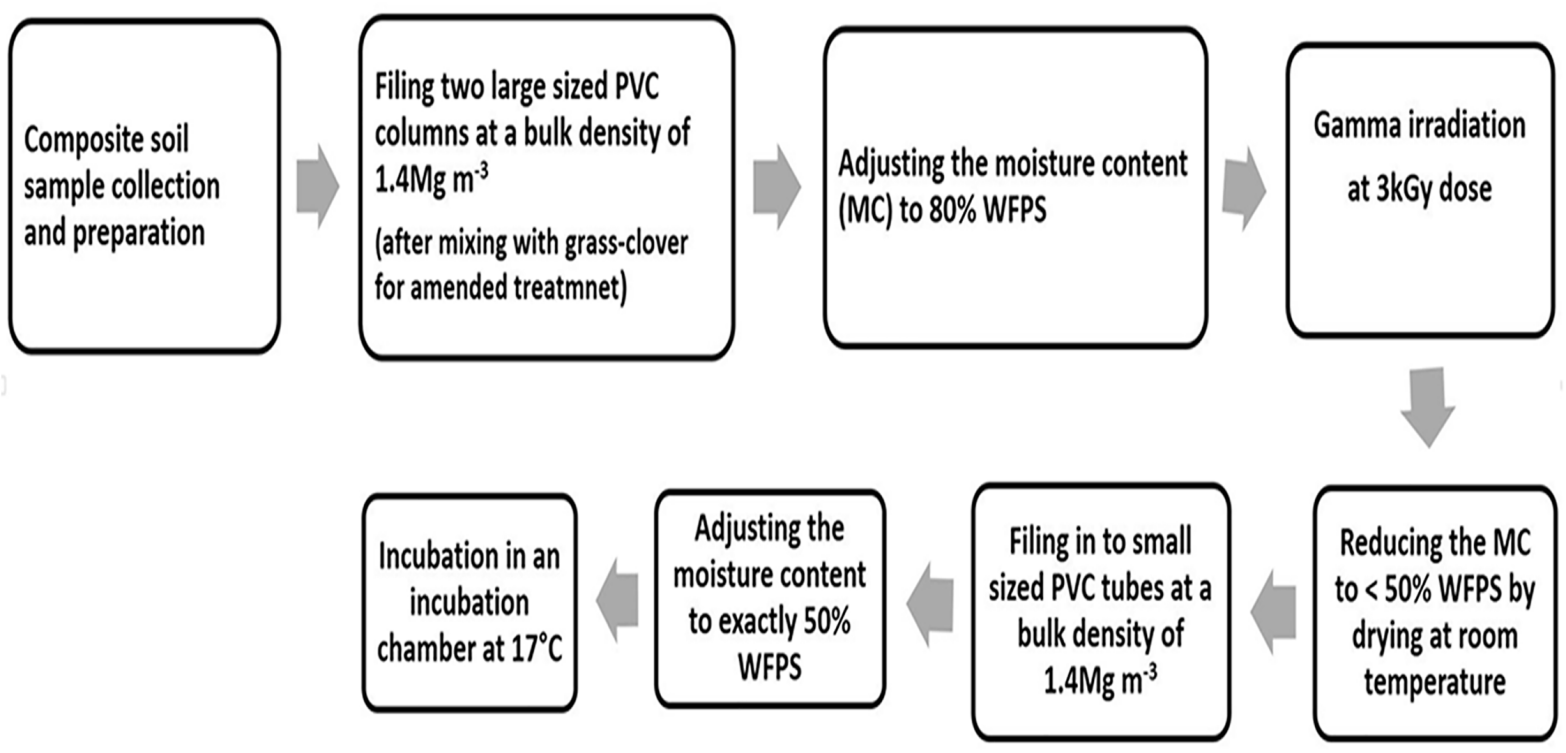
Schematic representation of the procedure followed to prepare the composite soil sample for gamma irradiation and incubation. Large sized PVC tubes were used for gamma irradiation and small sized cores for incubation.

Two of the PVC columns (one amended and one unamended) were prepared for gamma irradiation, a technique that removes all nematodes and other soil fauna without significantly altering the microbial community (bacteria and fungi) as described in detail in [19] and [20] (Fig 1). Briefly, the moisture content of the soil in each PVC column was adjusted to 80% water-filled pore space (WFPS) by adding demineralized water and left overnight to let the water uniformly distribute throughout the column. The two PVC columns were subjected to a 3 kGy dose of gamma irradiation at the Synergy Health sterilizing company, Etten-Leur, The Netherlands. The exact dose applied to each PVC column was measured with dosimeters placed at the top, middle and bottom of each PVC column and the average dosimeter reading in kGy was 3.07±0.34 (n = 6 ± s.d). The non-irradiated soil in the remaining two PVC columns (which were used as control) was kept in a cold room (4°C) for a day. The moisture content of the samples which was 80% WFPS at the time of irradiation was reduced to slightly lower than 50% WFPS immediately after irradiation by spreading the soil from each PVC column separately at room temperature.

### 2.2 Treatments and experimental setup

A total of 18 experimental units (six treatments in three replicates) were prepared by filling small sized PVC cores (7.5 x 7.5 cm) with 400 g of the respective amended and unamended soil (6 cores with control soil and 12 cores with irradiated soil) and gently compacted to a bulk density of 1.4 Mg m^-3^. Half of the PVC cores with irradiated soil (n = 6) were reinoculated with concentrated nematode suspensions extracted from the same amount of fresh soil (i.e., from 400 g fresh soil). Inoculation was done by dripping nematode suspension (about 10 ml) on the soil, with a separate pipette per core, and by gently mixing before filling the PVC cores. Appropriate amounts of demineralized water were added into each core to obtain a moisture content of exactly 50% WFPS just before the incubation experiment was started, and kept constant by weighing the tubes and adding demineralized water as needed. All the samples were then incubated in an incubation chamber at 17°C for six months. At the end of the incubation, the following parameters were determined: mineral N, microbial biomass carbon (C_mic_), phospholipid fatty acids (PLFA), dehydrogenase activity, and betaglucosidase activity. The following treatments were compared with and without grass-clover amendment: 1) unirradiated fresh soil (CTR), 2) irradiated and without nematodes (-Nem) and 3) irradiated and reinoculated with nematodes (+Nem).

### 2.3 Nematode extraction and identification

Nematode extraction, counting and identification was performed according to the procedure explained in detail in [19]. Briefly, free-living nematodes were extracted from 100 g moist soil (CTR and +Nem samples) using an automated zonal centrifuge machine which separates all free-living nematodes from mineral and most organic soil constituents based on their specific density [21].

After extraction, the nematode suspension was poured onto a sieve covered with a standard nematode Cotton Wool filter paper (223 mm pore size, HYGIA rapid) for 72 h at room temperature (±20°C) according to [22]. All viable nematodes that passed through the filter were then collected, concentrated and counted using a dissecting microscope (40x magnification). Mass fixation was carried out by adding hot 4% formaldehyde (65°C) at a ratio of 1:2 (5 ml nematode suspension and 10 ml formaldehyde) and cooling immediately in cold water according to the procedure by [22]. After six weeks of storage at room temperature, the formaldehyde was tapped off carefully until 1ml of the nematode suspension remained into which glycerin was added in 1:1 ratio. Slides were made for each sample and at least 125 individual nematodes were identified from each slide to family or when possible to genus level based on [23]. Nematode trophic groups were assigned to bacterivores, fungivores, omnivores-carnivores, and herbivores according to [24]. However, *Filenchus* was considered as fungivorous as suggested by other authors [19, 25] and the remaining genera of the Tylenchidae family considered as root feeders.

Nematode counting and identification were done at the start of the experiment for each replicate of the CTR and +Nem samples of the amended treatment in order to check whether reinoculation was successful both in terms of nematode abundances and diversity in comparison to the unirradiated CTR. At the end of the experiment, the abundances of nematodes were determined for each treatment.

### 2.4 C mineralization

Each soil core was placed inside a glass jar that was sealed air tight. A small vial containing 15 ml of 0.5M NaOH was also placed on a mesh on top of the soil core in order to trap CO_2_ evolved from the soil. C mineralization was monitored during six months of incubation by measuring the CO_2_ trapped in the vial containing NaOH through back titrating the excess NaOH with 0.2M HCl after precipitating the carbonates with BaCl_2_. During and after titration, the jars were left open for about 3hrs to replenish the soil with oxygen. Throughout the incubation, CO_2_ was determined at 23 sampling points (each time from 3 replicates) spread over 6 months. Initially, CO_2_ was measured every day during the first 72 hours, and sampling intensity was gradually changed to every three days, every week and finally to every two weeks as the rate of C mineralization was decreasing.

The cumulative amount of CO_2_ produced in each treatment was calculated for both amended and unamended samples. The cumulative amount of CO_2_ produced from the added grass-clover amendment was determined by considering the simple differences between the cumulative CO_2_ evolved from the grass-clover amended treatments and the cumulative CO_2_ evolved from the equivalent treatment in the unamended soil. The cumulative C mineralized (C_t_) was then plotted against incubation time (t). C (_t_) was further fitted to a parallel first order plus zero order kinetic model using the Levenberg-Marquardt algorithm:

$$C\left(t\right)=C_{0}\left(1-{\text{e}}^{-\text{kf}*\text{t}}\right)+ks\;*\;t$$

This model assumes the existence of two pools of available C with different resistance against microbial degradation, namely an easily mineralizable pool (C_0_) that mineralizes according to first order kinetics at a rate of k_f_ (day^−1^), and a more resistant C pool that mineralizes according to zero order kinetics at a rate of k_s_ (μg CO_2_-C g^−1^ soil day^−1^) [26].

### 2.5 Microbial Biomass C and Phospholipid fatty acids (PLFA)

Microbial biomass carbon was determined at the end of the incubation by the fumigation extraction technique using the procedure by [27]. Changes in the structure of the microbial community were determined based on microbial membrane phospholipid fatty acids (PLFA) following a method explained in details by [28]. Briefly, 4 g of freeze-dried soil from each sample was sieved (2 mm) in order to homogenize and remove the root fragments and stones. Phospholipids were extracted from this freeze-dried soil and transformed into methyl esters (FAMEs). Finally, individual FAMEs were identified and quantified by Gas Chromatography-Mass Spectrometry (GC-MS) on a Thermo Focus GC combined with a Thermo DSQ quadrupole MS (Interscience BVBA, Louvain-la-Neuve, Belgium) in the electron ionization mode.

The sums of marker fatty acid concentrations for selected microbial groups were calculated as follows. For Gram-positive bacteria the sum of i15: 0, a15: 0, i16: 0, a16: 0, i17: 0 and a17: 0; for Gram-negative bacteria cy17: 0, cy19: 0, C16:1ω7, C16:1ω9; for the actinomycetes the sum of 10-methyl branched saturated fatty acids [29–31]. For the total bacterial community, in addition to Gram-positive and Gram-negative bacteria, the fatty acids 15:0, 17:0 were also included. For saprotrophic fungi the marker PLFAs 18:2ω6c and 18:1ω9, and for arbuscular mycorrhizal fungi (AMF) 16:1ω5c were considered [32]. The bacterial: fungal ratio was calculated as the sum of total bacterial marker fatty acids divided by the saprotrophic fungal marker fatty acids.

### 2.6 Enzymatic activities

Dehydrogenase and betaglucosidase enzyme activities were determined at the end of the incubation. Dehydrogenase activity was determined by using Triphenyltetrazolium Chloride (TTC) as a substrate following a procedure by [33]. Briefly, 2 ml of 3% TTC and 2ml of Tris-buffer pH 7.8 was added into vials containing 5 g moist soil. The samples were incubated for 24 h at 37°C and put on a linear shaker (125 rev min^-1^) for 2 h after adding 20 ml of methanol. The extractant was then filtered through Whatman no. 5 filter and color intensity of the filtrate was measured using a Varian Cary 50 spectrophotometer at 450 nm. The same procedure was followed for the control except that 4 ml of Tris-buffer was added instead of 2 ml TTC. Dehydrogenase activity was determined as a difference between the sample (with substrate) and the control for each sample.

β-glucosidase activity in the soil samples was determined by using *p-*nitrophenyl-β-D-glucoside as a substrate according to the procedure by [34]. One gram moist soil was weighed in triplicate in glass vials and 4 ml of modified universal buffer and 1ml of 25 mM *p-*nitrophenyl-β-D-glucoside was added, thoroughly mixed and incubated for 1h at 37°C. After incubation, 1ml of 0.5M CaCl_2_ and 4 ml Tris buffer pH 12 was added and immediately filtered through Whatman no. 5 filter paper. The same procedure was followed for the control samples except that the substrate was added after incubation. The color intensity of the filtrate was measured at 400 nm with a Varian Cary 50 spectrophotometer.

### 2.7 Mineral N

Nitrogen mineralization was determined by measuring the evolution of mineral nitrogen (NH_4_^+^ and NO_3_^-^) at the end of the incubation. Both NH_4_^+^ and NO_3_^-^ were measured from the same aliquot colorimetrically with a continuous flow auto analyzer (Chem-lab 4, Skalar 223 Analytical, Breda, The Netherlands), following the extraction of 30 g moist soil with 150 ml 1M KCl (1:5 ratio) after shaking for one hour.

### 2.8 Statistical analysis

The experiment followed a factorial design with two fixed factors. The first factor was the amendment and had two levels: grass-clover amended and unamended. The second factor nematode had three levels: i) non-irradiated fresh soil (CTR), ii) defaunated and not reinoculated (-Nem) and iii) defaunated and reinoculated with the entire free living nematode community (+Nem). Statistical analysis was conducted accordingly using a two-way analysis of variance (ANOVA) model after checking the assumptions of homoscedasticity and normality of all the variables. Data transformation (log or sqrt) was performed for the variables that violated the assumptions for ANOVA. Whenever the interaction between amendment and nematode treatment was non-significant (p>0.05), only the main effects were compared, and Fisher’s least significant difference (LSD) was used to analyze mean differences. However, when the interaction term was significant (p<0.05), one-way ANOVA model was fitted for each time-treatment combination and Tukey's method was used for the post hoc mean difference analysis. The PLFA data were further analyzed by Principal Component Analysis (PCA) based on the correlation matrix after checking the sampling adequacy using the Kaiser-Mayer-Olkin (KMO) test. All statistical analysis was performed using IBM SPSS Statistics 20 software (SPSS inc., Chicago, USA).

## Results

### 3.1 Nematode abundances and diversity

**Fig 2:**
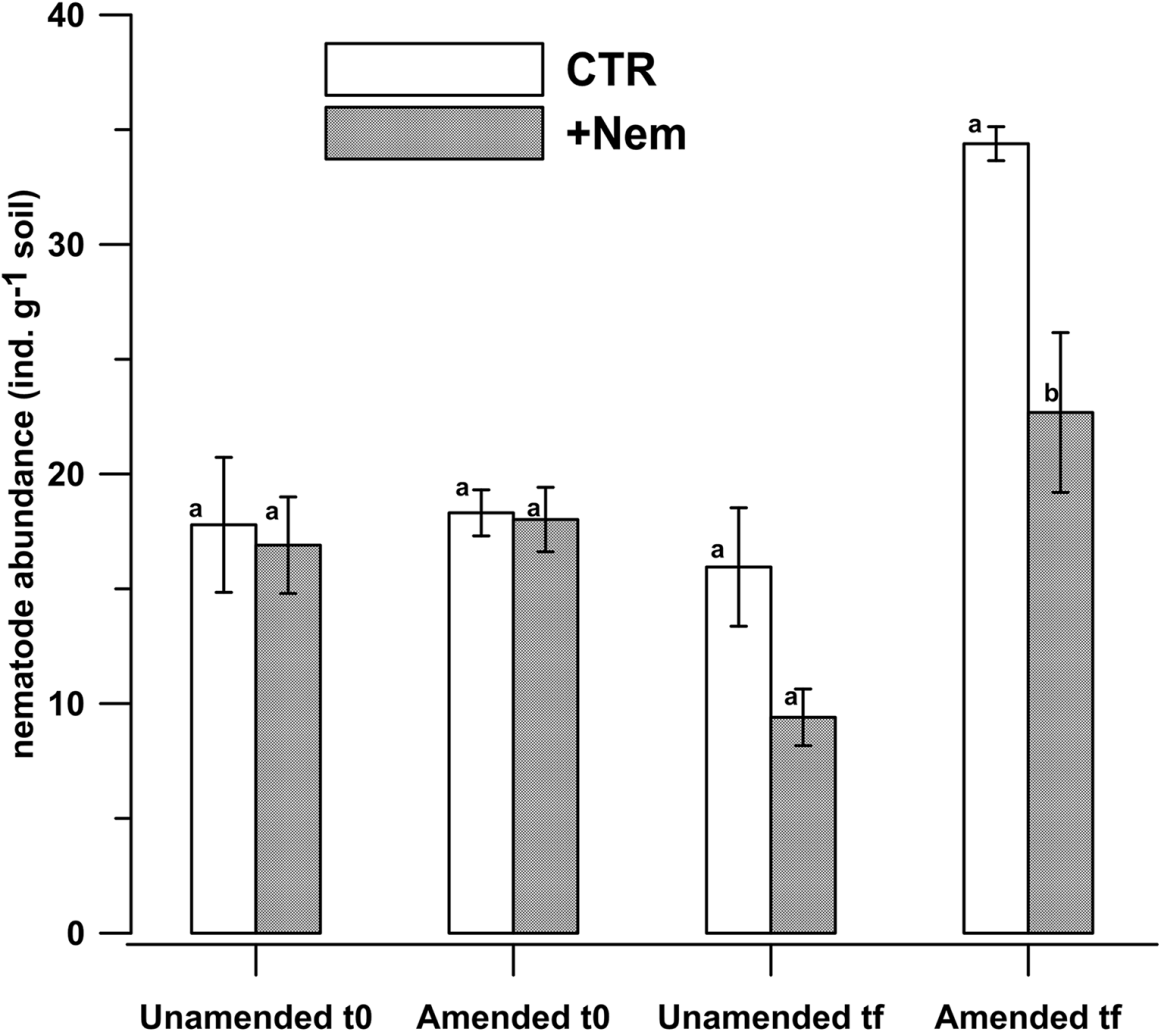
Nematode abundances (individual nematodes per gram of dry soil) in amended and unamended soil at the beginning (t0) and end (tf) of the incubation period. The error bars indicate standard error of the mean (n = 3) and different letters indicate significant statistical differences.

At the beginning of the incubation, all the nematode taxa identified in the unirradiated CTR samples were also found in the +Nem samples and no significant differences were found between the CTR and +Nem treatments in total nematode abundance (p = 0.894) and in abundances of herbivorous nematodes (p = 0.124), fungivorous (p = 0.358), bacterivorous (p = 0.217) and predators /omnivorous (p = 0.844) nematodes (S1 Table). At the end of the incubation, significantly lower abundances were found in +Nem samples than in the CTR in the amended samples only (p = 0.005), whereas in unamended samples the difference was not significant (p = 0.084) (Fig 2).

### 3.2 C mineralization

**Fig 3:**
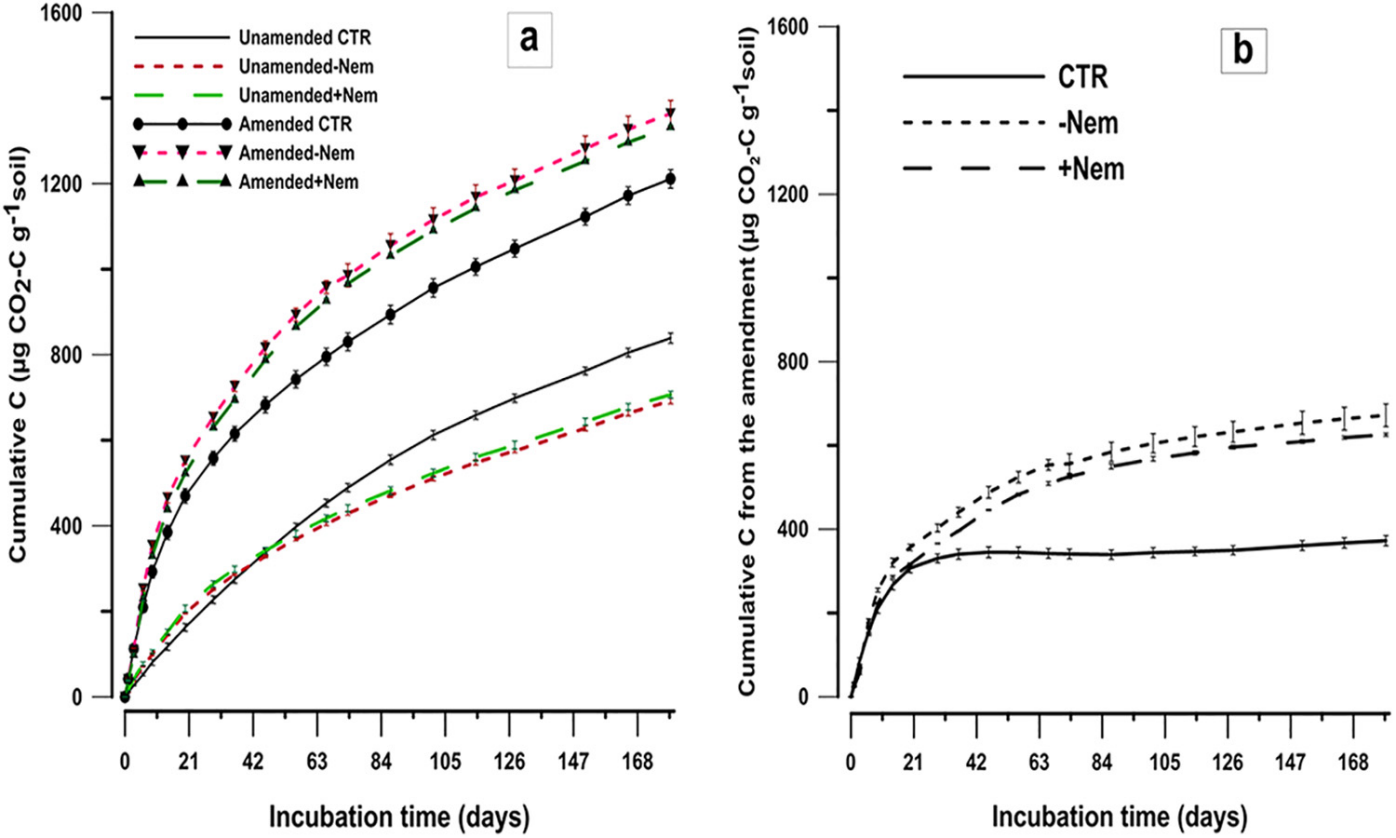
Cumulative C mineralization (μg CO_2_-C g^−1^ soil) as a function of the incubation time (a) and cumulative C mineralized from the grass-clover amendment (μg CO_2_-C g^−1^soil) over time calculated as a simple difference between each treatment of the grass-clover amended and unamended samples at each sampling date. Vertical bars indicate standard error of the mean (n = 3).

Cumulative C mineralization over time tended to be higher in +Nem than in–Nem treatments throughout the incubation period in unamended soil (Fig 3A). In amended soils, however, slightly higher cumulative C mineralization was found in–Nem than +Nem treatments. A significant interaction (p = 0.000) was found between amendment and treatment for cumulative C at the end of the incubation. However, no statistical significant differences were observed between +Nem and–Nem in both amended (p = 0.191) and unamended (p = 0.596) soils. In unirradiated CTR samples, cumulative C mineralization was lower than in both irradiated treatments (+Nem and–Nem) in amended soil throughout the incubation period (Fig 3A). The parameters of the first-plus-zero order kinetic model also followed a similar trend as the evolution of measured CO_2_-C in amended and unamended treatments (Tables 1 and 2). No statistical differences (p>0.05) were found between +Nem and -Nem treatments for all the model parameters (Table 1) and cumulative CO_2_-C estimated by the model at the end of the incubation, except K_f_ in amended samples. The CTR samples showed significantly lower C mineralized from the amendment than both +Nem (p = 0.000) and–Nem (p = 0.000) treatments.

**Table 1:** Parameters of the first-plus-zero order kinetic model fitted to the cumulative C mineralization data of each treatment (n = 3± standard error). Different letters refer to significant differences (p<0.05) between nematode treatments (Control = CTR, no nematodes = -Nem, and with nematodes = +Nem) in unamended or amended samples.

Factors/Treatments	C_0_ (μg C g^-1^soil)	k_f_ (day^-1^)	k_s_ (μg C g^-1^ soil day^-1^)
Unamended			
CTR	799.1±88.2a	0.009± 0.002a	0.91±0.35a
-Nem	286.2± 7.82b	0.035± 0.001b	2.27±0.06b
+Nem	290.9±6.21b	0.038±0.001b	2.32±0.02b
Grass-clover amended			
CTR	568.9±15.3a	0.060± 0.002a	3.63±0.06a
-Nem	740.3±21.13b	0.054±0.001b	3.54 ±0.15b
+Nem	570.2±6.41b	0.049±0.001c	3.39 ±0.04b
p (nematode*amendment)	*0*.*000*	*0*.*000*	*0*.*000*

C_0_ is the size of the readily mineralizable C pool; k_s_ and k_f_ are the mineralization rates of the slow and fast mineralizable C pools, respectively.

**Table 2:** Mean values (n = 3± standard error of the mean) of Cumulative CO_2_-C mineralized (model predicted values), microbial biomass C, and respiration rates at the end of the incubation in the amended and unamended soil. Different letters refer to significant differences (p<0.05) between nematode treatments in amended or unamended samples.

Factors/Treatments	Cumulative mineralized C (μg CO_2_-C g^-1^ soil)	Microbial biomass C (μg C g^-1^ soil)	Respiration rate (μg CO_2_-C g^-1^soil day^-1^)
**Unamended**			
CTR	865.8±12.20a	241.24±11.06a	4.49a
-Nem	723.6±7.2b	128.43±26.59b	3.72b
+Nem	739.1± 9.0b	172.51±6.9b	3.79b
**Grass-clover amended**			
CTR	1270.5±23.00a	198.00±5.79a	6.45a
-Nem	1423.8±33.70b	230.72±8.37a	7.25b
+Nem	1388.7±5.3b	195.20±10.49a	7.07b
p (nematode*amendment)	*0*.*000*	*0*.*001*	*0*.*000*

Net C mineralization from the grass-clover amendment, calculated as the simple differences in each treatment between amended and the corresponding unamended soil, tended to be higher in -Nem treatments throughout the incubation (Fig 3B). At the end of the incubation period, 48.7% (±1.93), 45.3% (±0.37) and 26.9% (±0.99) of the amended C was mineralized in -Nem, +Nem and CTR treatments, respectively. The net cumulative C mineralized from the amendment at the end of the incubation was not statistically different (p = 0.157) between +Nem and-Nem treatments.

### 3.3 Microbial Biomass C and community structure

A significant interaction (p = 0.01) was found between nematode treatment and amendment on C_mic_. The microbial biomass C showed no significant differences between +Nem and -Nem treatments both in unamended (p = 0.059) soil and in grass-clover amended (p = 0.087) soils (Table 2). The CTR samples, however, showed significantly higher C_mic_ than both–Nem and +Nem samples in unamended samples only (p = 0.000). In amended samples, no significant differences were found between the CTR and +Nem (p = 0.886) and CTR and–Nem treatments (p = 0.112).

**Fig 4:**
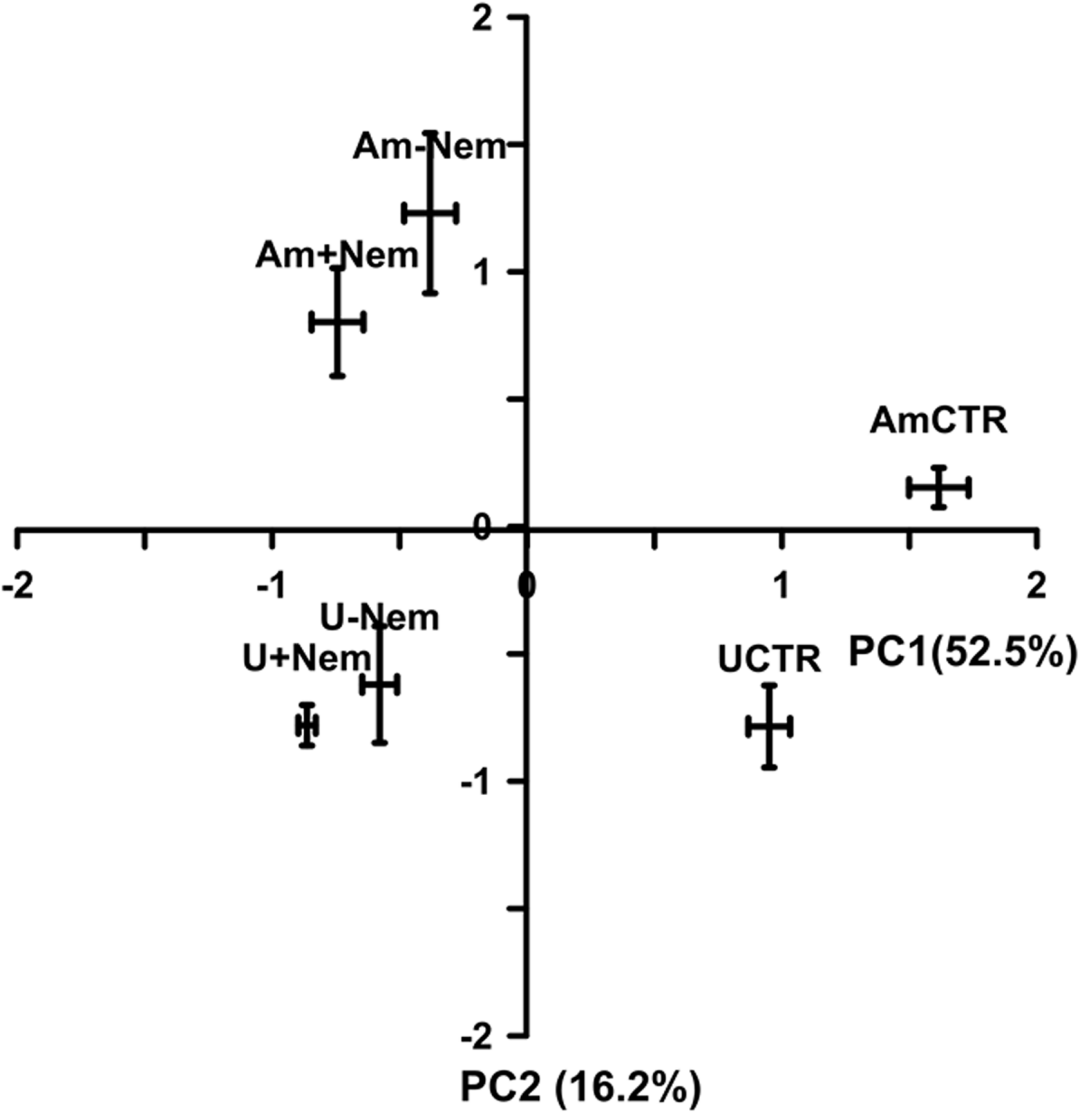
PLFA mean scores (n = 3) with standard error of the mean for the CTR,-Nem and +Nem in grass-clover amended (Am) and unamended (U) soil at the end of the incubation as analyzed by the principal component analysis.

There was no significant interaction between the nematode treatment and amendment on total PLFAs (p = 0.058) and signature PLFAs (Table 3). No significant differences were found between -Nem and +Nem treatments regardless of amendments in total PLFA (p = 0.273) and between amended and unamended treatments in the concentration of signature PLFAs of each of the microbial groups (Table 3). PCA analysis showed some differences between grass-clover amended and unamended treatments, and also between CTR and irradiated treatments (-Nem and +Nem). PC1 which explained 52% of the total variation clearly separated the CTR from both irradiated samples (Fig 4). PC2 that explained 16% of the total variation clearly separated the amended from the unamended samples regardless of treatments. PC1 is highly loaded with biomarker PLFAs of several microbial groups such as actinomycetes, G+ and G- bacteria and fungi, while PC2 is mainly loaded with non-signature fatty acids (C18:0 and C20:0) and a few signature PLFAs of bacteria and fungi (S2 Table).

**Table 3:** Mean concentrations of selected biomarkers for major microbial groups in nmole g^-1^ dry soil with standard error of the mean (n = 3 ± SE). The p values are based on two-way ANOVA model and show no significant differences between the levels of each factors.

Factors/treatments	Total PLFA	Gram+ bacteria	Gram-bacteria	Actinomycetes	Fungi	B:F ratio
Amendment						
Unamended	20.62±1.4a	4.60±0.68a	6.03±0.72a	1.89±0.39a	1.57±0.24a	8.30±0.85a
Grass-clover amended	24.14±1.1b	4.83±0.65a	6.24±0.68a	2.12±0.36a	1.77±0.24a	7.78±1.74a
Nematode						
CTR	27.01±0.87a	4.51±0.39a	5.93±0.43a	1.83±0.22a	1.59±0.15a	8.01±0.95a
-Nem	20.53±1.12b	5.02±0.79a	6.48±0.82a	2.20±0.49a	1.88±0.26a	7.58±2.05a
+Nem	19.58±0.86b	4.61±0.76a	6.00±0.81a	1.99±0.39a	1.54±0.27a	8.54±2.07a
*p (nematode)*	*0*.*00*	*0*.*61*	*0*.*72*	*0*.*65*	*0*.*32*	*0*.*18*
*p (amendment)*	*0*.*00*	*0*.*62*	*0*.*74*	*0*.*47*	*0*.*31*	*0*.*21*
p (nematode*amendment)	*0*.*55*	*0*.*94*	*0*.*21*	*0*.*98*	*0*.*88*	*0*.*56*

### 3.4 Enzymatic activities

No significant interaction was found between nematode treatment and amendment on both dehydrogenase (p = 0.091) and betaglucosidase (p = 0.117) activities. No significant differences were also found in dehydrogenase (p = 0.507) and betaglucosidase (p = 0.111) activities between +Nem and–Nem treatments regardless of the amendment (Table 4). However, CTR samples showed significantly higher enzymatic activities than both +Nem (p = 0.000) and -Nem (p = 0.003) treatments. Grass-clover amendment did not significantly increased dehydrogenase (p = 0.71) activities, but increased betaglucosidase (p = 0.000) activities compared to the unamended samples regardless of the nematode treatments.

**Table 4:** Enzymatic activities and mineral N concentration at the end of the six months incubation period. Significant interactions were found between the two factors amendment and nematodes. Different letters indicate significant differences (p<0.05) between the levels of the main factors.

Factors/Amendment	Dehydrogenase activity (μg TPF g^-1^ day^-1^)	Betaglucosidase activity (μg PNP g^-1^ day^-1^)	NH_4_^+^-N (mg kg^-1^soil)	NO_3_^-^-N (mg kg^-1^ soil)	Total mineral N (mg kg^-1^ soil)
Amendment					
Unamended	7.76±2.0a	52.69±2.3a	0.08±0.8a	93.90±3.5a	93.99±3.6a
Grass-Clover amended	8.46±1.3a	60.33±3.9b	1.32±0.6a	203.86±10.54b	205.20±10.56b
Nematode					
CTR	12.79±2.10a	66.54±2.94a	0.20±0.14a	132.83±21.69a	133.05±21.79a
-Nem	6.46±1.48b	54.36±3.51b	1.50±0.95a	143.43±23.62a	144.95±24.34a
+Nem	5.09±0.79b	48.63±1.89b	0.40±0.28a	170.37±30.53b	170.78±30.62b
*p (amendment)*	*0*.*71*	*0*.*02*	*0*.*08*	*0*.*00*	*0*.*00*
*p (nematode)*	*0*.*01*	*0*.*00*	*0*.*22*	*0*.*01*	*0*.*01*
p (nematode*amendment)	*0*.*09*	*0*.*12*	*0*.*13*	*0*.*07*	*0*.*09*

### 3.5 Mineral nitrogen

No significant interactions were found between nematode treatment and amendment on NH_4_^+^-N (p = 0.129) and NO_3_-N (p = 0.073) concentration at the end of the incubation. +Nem treatments resulted in significantly higher NO_3_-N (p = 0.011) and total mineral N (p = 0.015) concentration than–Nem treatments regardless of the amendment (Table 4). No significant differences were found in NH_4_^+^-N between nematode treatments, but grass-clover amended treatments resulted in slightly higher NH_4_^+^-N (mean difference 1.24 mg kg^-1^, p = 0.047) compared to unamended soil regardless of the nematode treatments.

## Discussion

The primary objective of this experiment was to quantify the collective contribution of the total free-living nematode communities to C mineralization from soil organic matter and grass-clover amendments over a six-month incubation. This was determined by simple differences in CO_2_-C mineralized between defaunated samples reinoculated with the entire free living nematode community (+Nem) and without nematodes (-Nem), as the only difference between the two treatments was the presence or absence of nematodes. CTR treatments comprise not only nematodes, but all other fauna. Thus, we did not compare it with +Nem treatments which have no other fauna except nematodes. The purpose of the CTR treatment of this experiment was to get background information on the field conditions and to evaluate the representativeness of the setup, particularly in terms of the reconstruction of the nematode community.

### 4.1 Validity and representativeness of the experimental approach

Nematode respiration is regulated by several biotic and abiotic factors such as the microbial biomass, the weight, size and growth stage of nematodes, the turnover rate of nematodes, food availability (mainly C and N), and ambient temperature [11, 18, 35]. Thus, experimental setups should allow all these interactions to occur as much as possible for a realistic determination of their role in soil heterotrophic respiration. In this experiment, we kept all these factors as representative as possible to the field conditions, particularly interactions between and within the indigenous microbial and the entire free living nematode communities.

In contrast to the previous experimental studies that comprised only a few selected species of bacterial feeding nematodes and bacteria (2, 18, 36), the current study includes all feeding groups of nematodes (S1) and indigenous microbial communities (Table 3). The indigenous microbial communities were left largely unaffected by applying low doses of gamma irradiation as reported in previous studies [19, 20, 28, 36]. High doses of gamma irradiation (e.g. 25 kGy) have normally been used to completely eliminate bacteria and fungi from the soil sample (e.g. [37]. In our experimental approach, we applied low doses (3 or 5 kGy depending on the moisture content of the soil) and removed nematodes and other fauna while leaving the microbial communities largely unaffected [19, 20, 28, 36]. In this way we avoided the need for extraction and reinoculation of the microbial communities.

Regarding the nutrient availability, grass-clover amended samples received an additional 1398 and 130 μg C and N g^-1^ dry soil, respectively, from the amendment, showing C was not limiting. Nitrogen, an element that often limits the microbial activity in terrestrial ecosystems, was present in high concentration and was significantly higher in amended than in unamended soil (Table 4) showing microbes were not nutrient limited in amended soil.

Despite the strong similarities in nematode and microbial communities, there are clear differences between the CTR and +Nem treatments. The CTR samples comprise the full diversity and abundance of soil fauna, including e.g. protozoa, mites and Collembola, whereas no fauna were present in +Nem except a limited abundance of protozoa who survived the irradiation and the reinoculated nematodes. Although the effect of gamma irradiation on soil physical, chemical and biological properties is relatively small (certainly compared to other sterilizing techniques), there certainly remain some effects such as nutrient flushes which create a difference between +Nem and CTR treatments.

### 4.2 C mineralization in amended and unamended soil

In contrast to our hypothesis, the presence of the entire free living nematode community did not significantly stimulate C mineralization neither from the native soil organic matter (SOM) nor from the added OM, although +Nem samples showed slightly higher effects (about 2%) at the end of the six months incubation in unamended soils. When assessing the effects of nematodes in a complex system such as soil with countless interactions, preferably a set of multiple related parameters should be used rather than relying on a single parameter. In both amended and unamended soils, the microbial biomass C, which regulates C mineralization, and the activities of dehydrogenase and betaglucosidase, which indicate microbial respiration and organic matter breakdown [33, 38], also showed no significant differences between +Nem and -Nem treatments (Table 4). Moreover, the parameters of the first-plus-zero order model generally resulted in no significant differences in mineralizable C, mineralization rates (both K_f_ and K_s_), and cumulative mineralized C between +Nem and -Nem treatments. These parameters additionally indicate that the presence of the entire free living nematodes was not significantly stimulating C mineralization regardless of the amendment.

Previous studies estimated that nematodes contribute between 0.3 to 2% of the total soil respiration in different forest ecosystems [11, 39]. The contribution of nematodes to C mineralization cannot be directly compared with the estimates mentioned above as the values were indirect estimates made based on oxygen consumption and estimates of RQ values. There are no previous comparable experimental setups that measured CO_2_ release from samples reinoculated with the entire free-living nematode community. A previous study reported cumulative CO_2_-C during 24 days of incubation in glucose amended and unamended soil samples using a single species of nematodes (*Mesodiplogaster*) and bacteria (*Pseudomonas*) [40]. This nematode species + *Pseudomonas* treatment increased C mineralization by 350 (50%) and 145 (27%) μg cumulative CO_2_-C g^-1^ soil over bacteria only treatments in glucose amended and unamended soil, respectively. In [40], the rate of total respiration was 43.5 and 28.1 μg CO_2_-C g^-1^ soil day^-1^ in the presence of nematodes in amended and unamended soils, respectively. This is in contrast to the current study, which found 7.07 and 3.79 μg CO_2_-C g^-1^ soil day^-1^ in the presence of nematodes in amended and unamended soils, respectively (Table 2). The rates in +Nem samples were comparable to the rate of respiration in the CTR samples, whereas in [40] there was no control soil to compare the results with.

In another experiment, the presence of *Mesodiplogaster iheritieri* or *Acrobeloides* sp. significantly increased C mineralization in glucose amended microcosms as compared to similar cores with bacteria only [9]. However, they used a very high density of nematodes, particularly for *Acrobeloides* sp. (96 ind. g^-1^ soil), a density which is highly unlikely to be present in field conditions. In the current experiment the abundances of the total nematodes in +Nem treatments of the grass-clover amended soil were 18 and 23 ind. g^-1^ soil at the start and end of the experiment, respectively. Presumably, the contribution of a single species of nematodes to C mineralization by feeding on a single food source (bacteria) is contrasting to the contribution of the entire nematode community in the presence of multiple food sources including native microflora.

### 4.3 Possible explanations for the insignificant effects of nematodes on C mineralization

The first possible explanation could be related to the mechanisms how nematodes are involved in C mineralization. One of the mechanisms through which nematodes increase microbial activities such as OM decomposition is by transporting microbes from a region of less or no available food to substrate rich microsites [6]. Given the availability of sufficient and relatively more uniformly distributed substrate and available nutrients in the amended microcosms in this experiment, microbes may not have benefited from the presence of nematodes. It has also been found that nematodes migrate towards ‘hot spots’ [41, 42], but their movement may be limited once they reach these ‘hotspots’ [43]. It has been suggested that, in the presence of a higher concentration of easily degradable C, the bacterial degradation may be sufficient to release the required amount of nutrients, particularly N, and the contribution of microbial grazers such as nematodes could be negligible [44]. A similar suggestion was also given based on findings in amended microcosms [45]. Our findings appear to support these hypotheses that in nutrient rich conditions (amended soil) the presence of nematodes may not significantly enhance OM decomposition and the subsequent nutrient mineralization. However, in situations of low nutrient availability, the production efficiency of nematodes may be drastically reduced, and respiration may even exceed assimilation [39]. The findings in the current experiment indicate that the contribution of the entire nematode community to C mineralization is not significant, regardless of the resource availability. Although the effect was not significant, reduction of production efficiency could be one of the reasons for the slight increase in OM decomposition in +Nem treatments in resource poor condition (unamended soil).

The second explanation may be related to the abundance and possible shifts in the community structure of both nematode and microbial communities. The contribution of each trophic group of nematodes to nutrient mineralization is different [46]. The available studies confirmed that some species of bacterivores significantly increase C mineralization as mentioned in section 4.2. In a recent study, [36] found a significant effect of nematodes on nutrient mineralization in bacterivores dominated bare microcosms compared to herbivores dominated planted microcosms, despite a twofold abundance in the latter treatment. Other reports indicated that there could be a shift in nematode community structure over time depending on the availability of resources [36, 47, 48]. In another experiment with clover amended soils, the abundance of nematodes significantly increased over time, and the community shifted to bacterivorous nematodes [49]. Given these evolutions in our previous findings [36, 49, 50], bacterivores were possibly dominating the nematode community in the current experiment as well. However, given the effect of nematodes on microbes is density dependent [51], the possible domination of bacterivorous nematodes only, may not bring significant effects unless they are present in sufficient numbers. Therefore, the low abundance of nematodes in the unamended soil could possibly be another reason for the insignificant effect of nematodes on OM decomposition.

The principal component analysis of PLFA data showed that there was a slight shift in microbial community structure, as illustrated in Fig 4. For instance, PC1 separated the CTR treatments from both +Nem and–Nem treatments regardless of the amendment. Given that the CTR samples comprise the full diversity of soil organisms, the composition of PLFAs in the CTR was expected to be different from the +Nem and -Nem treatments which contain PLFAs from the nematode and microbial communities only. However, no single microbial group dominated the CTR or the nematode treatments as indicated by the uniform loadings of PC1 (S2 Table) and further supported by the absence of significant differences in microbial groups between the nematode treatments (Table 3). PC2 separated amended from unamended treatments, but it accounted only for 16% of the total variation. The non-biomarker long chain saturated PLFAs such as C17:0 and C20:0 dominated the loadings of PC2, suggesting that organisms with these PLFAs were segregating the amended from the unamended treatments. The abundance of each microbial group generally tended to be higher in amended treatments than in unamended treatments (Table 3), indicating that the grass-clover amendment stimulated the entire microbial community, instead of shifting the microbial community structure.

Previous studies applied a suite of molecular approaches to study the effects of different levels of nitrogen on bacterial diversity and found a shift in bacterial community that likely led to a decrease in soil respiration at high N availability [52]. Nematodes have also been reported to change the structure of the bacterial community responsible for nitrification [53] and denitrification [54] in the soil. Possibly there might have been a shift in the microbial community in amended treatments that reduced respiration e.g. from oligotrophs to copiotrophs as reported by [52]. Such kind of changes in the taxonomic composition of a particular community is difficult to be detected using PLFA profiles.

## Conclusions

In this experiment, we studied the contribution of the entire free living nematode community to C mineralization in the presence of the native microbial communities and contrasting nutrient availabilities for the first time. Our data showed that the collective contribution of the entire free-living nematode community to C mineralization is small. The reinoculated treatments (+Nem) resembled the field soil (CTR) in microbial and nematode communities, but the former differed in terms of nutrient flush and mesofauna which made direct comparison with the CTR difficult. The nematode density in the current experiment was lower compared to previous experiments and/or significantly decreased over time, which could be one of the reasons for the lack of significant effects on C mineralization. We suggest that further similar studies are needed using higher nematode densities, including a more detailed analysis of the evolutions in the functional assemblage of microbial and nematode communities over time.

## Supporting Information

S1 Table Mean nematode abundances (ind. g^-1^ soil) and SE of the mean (n = 3) in unirradiated fresh soil (CTR) and irradiated and reinoculated soil (+Nem) at the start of the incubation experiment. (DOCX)

S2 Table Loadings of individual FAMEs in the principal components (PC1 and PC1) of both amended and unamended samples after analysis of principal component (PCA). (DOCX)
