# Supplementary material for: Quantifying the Contribution of Entire Free-Living Nematode Communities to Carbon Mineralization under Contrasting C and N Availability
Source: PLoS One. 2015 Sep 22;10(9):e0136244. doi: 10.1371/journal.pone.0136244 (PMC4579140; doi:10.1371/journal.pone.0136244)
Supplement: S1 Table — (DOCX) [file pone.0136244.s001.docx]

| Family/Genus | Feeding groups | Mean abundance  (individuals g**^-1^** soil) ± SE | |
| --- | --- | --- | --- |
|  |  | CTR | Reinoculated |
|  |  |  | (+Nem) |
| *Coslenchus* | Plant and root | 0.48±0.01 | 1.56±0.23 |
| Dolichodoridae | Plant and root | 0.24±0.06 | 0.70±0.09 |
| Heteroderinae | Plant and root | 1.95±0.19 | 1.28±0.21 |
| Tylenchidae | Plant and root | 2.08±0.11 | 2.09±0.73 |
| *Paratylenchus* | Plant and root | 0.59±0.08 | 1.47±0.41 |
| *Meloidogyne* | Plant and root | 0.83±0.02 | 0.33±0.18 |
| *Pratylenchus* | Plant and root | 0.18±0.04 | 0.18±0.09 |
| *Psilenchus* | Plant and root | 0.12±0.00 | 0.22±0.11 |
| ***All herbivores*** |  | **6.47±0.08a** | **7.84±0.5a** |
| *Filenchus* | Fungivorous | 0.18±0.04 | 0.40±0.03 |
| Aphelenchoides | Fungivorous | 0.11±0.09 | 0.24±0.11 |
| *Aphelenchus* | Fungivorous | 0.34±0.28 | 0.49±0.07 |
| ***All fungivores*** |  | **0.64±0.51a** | **1.18±0.25a** |
| Panagrolaimidae | Bacterivorous | 0.06±0.05 | 0.37±0.31 |
| *Plectus* | Bacterivorous | 0.06±0.05 | 0.08±0.08 |
| Cephalobidae | Bacterivorous | 4.68±0.21 | 3.49±0.38 |
| *Pristionchus* | Bacterivorous | 0.11±0.09 | 0.49±0.07 |
| Rhabditidae | Bacterivorous | 5.93±0.32 | 4.30±0.48 |
| ***All bacterivores*** |  | **10.84±0.88a** | **8.69±0.94a** |
| *Seinura* | Predator | 0.00±0.00 | 0.04±0.04 |
| Steinernema | Bacterivorous and Enthomopathogenic | 0.00±0.00 | 0.04±0.04 |
| Nygolaimidae | Carnivores/predators | 0.08±0.02 | 0.05±0.05 |
| Dorylaimidae | Omnivorous | 0.36±0.20 | 0.17±0.11 |
| ***All predators/omnivores*** |  | **0.36±0.25a** | **0.3±0.18a** |
| **Total** |  | **18.31±1.00a** | **18.02±1.40a** |
