# Supplementary material for: Quantifying the Contribution of Entire Free-Living Nematode Communities to Carbon Mineralization under Contrasting C and N Availability
Source: PLoS One. 2015 Sep 22;10(9):e0136244. doi: 10.1371/journal.pone.0136244 (PMC4579140; doi:10.1371/journal.pone.0136244)
Supplement: S2 Table — (DOCX) [file pone.0136244.s002.docx]

| PLFA | PC1 |  | PLFA | PC2 |
| --- | --- | --- | --- | --- |
| 10Me16:0 | 0.93 |  | C20:0 | 0.93 |
| aC15:0 | 0.93 |  | C18:0 | 0.91 |
| cyC19:0 | 0.93 |  | iC17:0 | 0.86 |
| 10Me1:08 | 0.92 |  | C18:3C9 | 0.73 |
| C18:1ω7 | 0.92 |  | C17:0 | 0.69 |
| C18:1ω9c | 0.92 |  | C15:0 | 0.63 |
| cy19:0new | 0.91 |  | C20:2ω6 | 0.60 |
| cyC17:0 | 0.88 |  | C18:1ω9t | 0.59 |
| iC15:0 | 0.87 |  | C20:1C11 | 0.58 |
| C16:1ω7 | 0.86 |  | C16:0 | 0.56 |
| iC14:0 | 0.83 |  | C20:3ω6 | 0.54 |
| C18:3C6,9,12 | 0.83 |  | C18:2C9 | 0.53 |
| C20:5 | 0.82 |  | C23:0 | 0.50 |
| 10MeC:170 | 0.82 |  | C22:0 | 0.48 |
| aC17:0 | 0.82 |  | C21:0 | 0.42 |
| cy17:0new | 0.81 |  | iC16:0 | 0.40 |
| iC16:0 | 0.80 |  | i19:0 | 0.39 |
| i19:0 | 0.78 |  | aC17:0 | 0.38 |
| C14:0 | 0.77 |  | 10Me17:0 | 0.33 |
| C20:4 | 0.71 |  | C14:0 | 0.28 |
| C16:1ω5 | 0.69 |  | C18:1C13 | 0.27 |
| C16:0 | 0.68 |  | C24:0 | 0.22 |
| C15:0 | 0.66 |  | cyC19:0 | 0.22 |
| C18:2C9,12 | 0.65 |  | C20:4 | 0.20 |
| C17:0 | 0.65 |  | C18:1ω9c | 0.18 |
| C22:0 | 0.46 |  | C16:1ω5 | 0.17 |
| C18:3C9,12,15 | 0.41 |  | cy19:0new | 0.17 |
| C24:0 | 0.26 |  | C24:1ω9 | 0.14 |
| C20:1C11 | 0.25 |  | iC14:0 | 0.14 |
| aC16:0 | 0.25 |  | C18:3C6 | 0.12 |
| C20:3ω6 | 0.24 |  | C10:0 | 0.09 |
| C24:1ω9 | 0.21 |  | C11:0 | 0.09 |
| iC17:0 | 0.21 |  | C20:5 | 0.08 |
| C18:0 | 0.20 |  | C16:1ω7 | 0.07 |
| C23:0 | 0.18 |  | iC15:0 | 0.07 |
| C20:2ω6,9 | 0.15 |  | cy17:0new | 0.07 |
| C18:1C13 | 0.12 |  | 10Me16:0 | 0.06 |
| C18:1ω9t | 0.10 |  | aC15:0 | 0.06 |
| C20:0 | 0.10 |  | C18:1ω7 | 0.05 |
| C11:0 | 0.07 |  | cyC17:0 | 0.05 |
| C10:0 | 0.02 |  | 10Me18:0 | 0.01 |
